# New Biomedical Technologies and Strategies for Prevention of HIV and Other Sexually Transmitted Infections

**DOI:** 10.1155/2016/7684768

**Published:** 2016-09-15

**Authors:** Bonaventura C. T. Mpondo

**Affiliations:** Department of Internal Medicine, College of Health and Allied Sciences, The University of Dodoma, Dodoma, Tanzania

## Abstract

Sexually transmitted infections remain to be of public health concern in many developing countries. Their control is important, considering the high incidence of acute infections, complications and sequelae, and their socioeconomic impact. This article discusses the new biomedical technologies and strategies for the prevention of HIV and other sexually transmitted infections.

## 1. Introduction

Sexually transmitted/transmissible infections (STIs) also referred to as sexually transmitted diseases (STDs) or venereal diseases (VDs) are infections that can be transmitted from one person to another through sex (vaginal intercourse, anal sex, or oral sex). Sexually transmissible infections (STIs) are common [1]. A number of these infections in addition to being acquired as a result of sexual contact can also be acquired by other means, such as contact with blood or blood products.

STIs have major demographic, economic, social, and political impact on many populations, particularly sub-Saharan Africa and Asia [1]. In 1993, the World Bank estimated that STIs (excluding HIV) were the second most common cause of healthy life lost among women aged 15–44 years after maternal morbidity and mortality [2]. It is estimated that up to 1 million people are newly infected with STIs daily. The pattern and distribution of STIs vary considerably between countries, but also within countries there are significant geographical variations.

Despite sharing a common route of transmission, STIs constitute a group of diverse infections with multiple causative agents including viruses (e.g., human immunodeficiency virus, HIV; herpes simplex virus, HSV; human papilloma virus, HPV; and hepatitis B virus, HBV), bacteria (e.g.,* gonococcus*,* Chlamydia*, and syphilis), and protozoa (e.g., trichomoniasis). Preventive strategies for these infections should be based on the involved pathogens [3]. The majority of the preventive measures for HIV and other STIs that have been used for many years were dependent on male controlled methods despite the fact that more than 90% of the infections worldwide result from heterosexual sex [4]. Most of these traditional methods have been shown to poorly prevent HIV and other STIs [5, 6]. There are several new technologies and strategies for prevention of HIV and other STIs. This minireview aims at discussing new technological advances and new strategies for the prevention of HIV and other STIs. The article gives a synopsis of key papers and studies on prevention of STIs.

## 2. Proven New Technologies for STI Control

### 2.1. HPV Vaccine

Human papilloma viruses (HPV) are DNA viruses that potentially infect basal epithelial cells of the skin or mucous membranes. There are about 100 different identified genotypes of HPV; out of these 40 are implicated in genital infection [7]. Genital HPV genotypes are classified into high risk types (16/18/31/33/35/39/45/51/52/56/58/66/68) [8] when associated with cervical cancer and low risk types (6/11/40/42/43/44/54/61/72) when only associated with condyloma acuminata [9]. HPV infection can occur at any age. There are reports of HPV infection in healthy young children. The prevalence of HPV has been shown to be inversely related to age in many countries, but in some countries, which are very poor, the prevalence has been shown to be high across all age groups [10]. Genital HPV is transmitted primarily by skin-to-skin contact, usually during sexual intercourse. Virtually all cervical cancer cases arise from genital HPV infection [11] which necessitates the prevention of genital HPV.

HPV vaccines are prepared from protein shells called virus-like particles through recombinant gene technology. These are noninfectious because they contain no viral biological product or DNA. Most of the currently available vaccines are bivalent and designed to target HPV genotypes 16 and 18 which are the genotypes associated with most cases of cervical cancer. There are also quadrivalent vaccines which have a protective effect against the low risk viral genotypes, 6 and 11.

HPV vaccines provide prophylaxis by preventing infections and consequent disease in at-risk individuals. Available data show that there is no protective effect among women who have already been exposed to HPV 16 and HPV 18 before vaccination. The vaccines however have shown to be highly effective in the prevention of HPV infection in clinical trials [12]. In clinical trials, nearly 100% of vaccinated women develop detectable levels of antibodies against each HPV genotype present in the vaccine [13, 14]. There is also some degree of cross-protection against HPV 31 and HPV 45 which are closely related to HPV 16 and HPV 18, respectively. Studies show that the vaccine continues to be immunogenic and well tolerated after 9 years of follow-up [15]. In vivo studies have shown that the antibody produced by the vaccine prevents virion binding to the cervicovaginal basement membrane [16].

The Advisory Committee on Immunization Practices (ACIP) of the Centers for Disease Control and Prevention (CDC) recommends that routine HPV vaccination should be initiated at the age of 11 or 12 years [17]. It also recommends that the vaccine should be given as a 3-dose series, the second dose being administered at least 1 to 2 months after the first dose and the third dose at least 6 months after the first dose. The recommendation is that the series can be started at the age of 9 years. For females or males who have not been vaccinated previously or who have not completed the 3-dose series, it is recommended to administer the vaccine at the age of 13 through 26 years and 13 through 21 years, respectively.

The current HPV vaccines however have several shortcomings. The main one is their negligible prophylactic effect against other oncogenic genotypes that are not targeted by the vaccine. An effective vaccine therefore should be multivalent and take into account all the high risk HPV genotypes. However these would be more expensive than the existing ones. Overall the uptake of the vaccine has been low due to relatively high cost, parental concerns, lack of provider recommendations, and limited enthusiasm from some health care providers.

### 2.2. HPV DNA Testing

Invasive cervical cancer is rated as the third most common tumor in women throughout the world [18]. The incidence of and mortality from cervical cancer vary across the globe; estimates show that more than 85% of invasive cervical cancers occur in low to middle income countries [19]. Persistent infection with high risk genotypes of HPV, though not a sufficient cause, is considered a necessary cause [11].

Screening for cervical cancer using a cytology based test has been fundamental for decreasing the incidence and associated mortality in countries with wide screening coverage. In asymptomatic women, one of the most common cytological findings is atypical squamous cells of undetermined significance. This finding forms the boundary between normal and abnormal cytological findings. The use of a single cytological screen however has low sensitivity that can be as low as 50–60% [20]. Despite the high specificity of the test in ruling out cervical cancer among healthy women, the test requires repeated rounds of screening to detect cervical intraepithelial neoplasm (CIN) 3 lesions among women with cervical cancer [21, 22]. Management of these patients includes referral to costly and invasive colposcopy.

Recent studies have demonstrated that HPV DNA tests have higher sensitivity than cytological screening for the detection of CIN3 [2–25]. The negative predictive value (i.e., the reassurance of not developing CIN3 and invasive cervical cancer among those that have a negative screening test) is higher with the HPV DNA test than in cytology screening. This allows longer screening intervals which reduces the cost [26, 27]. There is sufficient evidence from studies to recommend the HPV DNA test for triaging women with atypical cytology and close monitoring after treatment of cervical intraepithelial neoplasm lesions [28]. Because of its high sensitivity, careful choices of which HPV types should be targeted and the threshold for a positive result are required to optimize the clinical specificity of HPV testing while maintaining its sensitivity for CIN3 detection.

## 3. Technologies in Experimental Stage

### 3.1. Microbicides: Vaginal and Rectal Gels

In sub-Saharan Africa, where a majority of world HIV cases are, women account for about 59% of all infected adults. The number of HIV-positive women aged between 15 and 24 years is threefold higher than that of their male peers representing 76% of HIV cases in that age group [29]. These figures demonstrate the vulnerability of women in acquiring HIV as compared to their male counterparts. Several factors contribute to the higher risk of acquiring HIV in women than in men. Apart from the known biological factors [30, 31], some sexual behavior patterns such as low marriage rates [32], partnering with older men, inconsistent condom use [33], multiple partners [34], and limited skills in negotiating safer sex put women at an increased risk of acquiring HIV. As a result, measures to prevent mucosal transmission of HIV to women are needed.

Topical microbicides were proposed approximately two decades ago [35]. The use of topical microbicides has an advantage because women can initiate and control its use unlike most of the other preventive measures. Research on developing potent microbicides is ongoing and several candidates are undergoing effectiveness trials to assess their impact in HIV prevention. Most of the tested microbicides however were found to have disappointing results in protection against HIV [36, 37]; some were found to be potentially harmful [38]. The microbicide trials network VOICE trial, which tested daily dosing of tenofovir (TFV) gel, oral TDF, and oral combination of tenofovir and emtricitabine (Truvada) in women of South Africa and east African countries of Uganda and Tanzania, found no significant HIV protective effect in any of the intervention groups [39]. However, drug level testing in these women showed very low adherence rates that could have influenced the findings [40]. Trials for microbicides for the prevention of nonulcerative STIs have also been disappointing [41].

Recently, research in developing microbicides for prevention of mucosal HIV transmission has focused on using antiretroviral agents in various formulations and dosing strategies. Tenofovir, a nucleotide analog antiretroviral, has been shown to be safe [42] with an overall HIV protective effect of 39% (54% in women with high adherence) and 51% against herpes simplex virus type 2 (HSV-2) [43]. Comparing the results of VOICE trial and results of the Centre for the AIDS Programme of Research in South Africa (CAPRISA) 004 trial, it is evident that adherence plays a critical role in the success or failure of ARV-based microbicides. Initially it was formulated for oral use [44], but, because of its efficacy, long half-life, and favorable safety profile, it was considered as an ideal drug for making microbicides [45]. Results of the (CAPRISA) 004 trial therefore demonstrate that TFV gel could potentially fill the HIV prevention gap especially for women who cannot negotiate safe sex. The results of this trial however need to be confirmed.

A rectal microbicide is a topical substance, which may be prepared in the form of a lube, anal douche, or a wash. Rectal microbicides are developed and tested to reduce a person's risk of acquiring HIV and other STIs through anal intercourse. Despite the fact that anal intercourse increases the risk of HIV infection by as much as 10 to 20 percent compared to vaginal intercourse [46], most research on microbicides has been focused on vaginal intercourse. Scientists have tried the use of vaginally formulated 1% TFV gel product for rectal protection; however, it was found to be neither safe nor acceptable for rectal use [47].

Rectal microbicides have been in clinical development for more than ten years. Studies in nonhuman primates have demonstrated that antiretroviral gels provide rectal protection [48, 49]. The results of a phase II trial (MTN 017) designed to evaluate the rectal safety, drug absorption, and acceptability of a reduced glycerin formulation of TFV gel which is a reformulated version of vaginal TFV gel as well as oral Truvada have been reported. MTN-017 found that reduced glycerin TFV gel was safe. Most side effects from study products were minor, and there were no significant differences in adverse events with the gel regimens (daily and on demand) compared to oral Truvada [50]. The results of MTN-017 support further studies on the use of reduced glycerine 1% TFV gel as a rectal microbicide for HIV prevention among men who have sex with men (MSM) and transgender women (TGW). A double blind randomized phase 2A study of dapivirine 0.05% gel applied rectally in HIV-1 seronegative adults (MTN-035) is currently in development stage.

### 3.2. Microbicides: Vaginal Rings

The fight against HIV/AIDS cannot be achieved with care and treatment alone. As the efforts in finding an effective vaccine remain unsuccessful, several alternative methods for preventing HIV have been proposed. As previously discussed, there is a need for female-controlled HIV prevention strategies which includes the development of vaginal microbicides: compounds designed to achieve a topical preexposure prophylaxis. However, as discussed, the microbicides which were studied did not appear to be very effective. There were also issues of adherence.

Vaginal rings typically sit near the cervix and deliver controlled release of a drug. The rings are coated with a nonnucleoside reverse transcriptase inhibitor dapivirine and replaced every month. In a recently published study which was conducted in multiple sites in sub-Saharan Africa (ASPIRE study), women who used the ring were 27 percent less likely to become infected with the HIV virus [51]. Another sister trial (The Ring Study) found that the ring was 31% effective [52]; women who used the ring were 31 percent less likely to become infected with the HIV virus. The efficacy increased with increasing level of adherence. In the ASPIRE study, when the scientists excluded data from 2 sites where many women were not returning for study visits or using the ring consistently, the ring reduced the risk of HIV infection by 37%. The open label extension studies for the two trials are underway.

### 3.3. Microbicides: Vaginal Tablets, Films, and Nanofibers

In order to achieve success in prevention of HIV among the women worldwide, there should be a diversity of microbicide delivery systems that takes into account the varied populations of women all over the world. A number of microbicide products are in various stages of their development; some of the microbicides being developed include films, nanofibers, and tablets [53] which have better drug delivery systems compared to vaginal gels.

Vaginal tablets are easily formulated and manufactured; however, they may leave a grainy residue in the vaginal cavity after dissolution. Vaginal films are also relevant for pericoital use [54]. They have demonstrated capability of delivering physicochemically diverse agents and exhibit enhanced product stability compared to vaginal gels that are semisolid. Polymeric films provide rapid drug release and bioadhesive properties that may increase retention time at the target tissue. The films have been investigated for mucosal and transdermal drug delivery. The use of nanoparticle encapsulation is also being investigated as a possible drug delivery system for a microbicide. Nanoparticles formulated from the biodegradable copolymer poly (lactic-co-glycolic acid) (PLGA) have been shown to be effective when used as a drug delivery system [55].

Animal studies on rapidly disintegrating vaginal tablets containing TFV either alone or in combination with emtricitabine demonstrated favorable vaginal tissue and fluid concentrations of both drugs [56, 57]. Currently, a phase I placebo controlled safety of vaginal tablets in HIV negative women is ongoing.

Another trial compared the safety, drug absorption, and drug distribution of dapivirine containing vaginal films and vaginal gels. The preliminary results of the trial (FAME 02) show that plasma levels of dapivirine were comparable across the film and gel arms, suggesting that both products can deliver drugs with similar efficacy [58]. There are also ongoing trials (FAME 04) on a cellulose-based film containing tenofovir (currently in a phase I) and on biodegradable electrospun nanofibers containing agents including tenofovir, griffithsin, or carrageenan with activity against HIV, HSV, and HPV.

### 3.4. HIV Vaccine

One of the highest priorities in the HIV pandemic response is to develop an efficacious and protective HIV vaccine. An efficacious and protective vaccine would be the best long term tool for the control of HIV infection. The search for an efficacious HIV vaccine has been an ongoing exercise for the past two decades; however results have been disappointing [59, 60].

The process of developing an HIV vaccine faces a number of scientific obstacles. The main obstacles include the high mutation rate of the virus resulting in high viral amino acid sequence variability [61] and the concern of causing an autoimmune phenomenon following cross-reactivity between viral and host proteomes [62]. Studies have shown that there is very high peptide identity pattern between HIV and humans which increases the risk of cross-reactivity in the course of HIV-1 immune responses [63, 64].

To develop a safe, effective, and universally acceptable vaccine, unique viral peptide signatures should be used to minimize potential for harmful collateral cross-reactions. However, the immune correlation and the quality and magnitude of the immune responses needed for a protective effective against HIV remain unclear. Typically, vaccine development is based on ability of the vaccine to induce immune responses involving both neutralizing antibodies and activation of cytotoxic T lymphocytes as it would occur in nature following an infection. An initial approach in vaccine development tried to use VaxGen's AIDSVax which is a recombinant form of glycoprotein 120 (gp 120) that forms part of the HIV envelop. However this vaccine did not show any protective effect against HIV because it failed to induce formation of broadly neutralizing antibodies [65].

The vaccine that has demonstrated some encouraging findings is the one that is being tested in Thailand. In the Thai clinical trial (RV 144), a combination vaccine composed of priming doses of Vcp1521, a recombinant canarypox viral vector, followed by a boosting dose consisting of both the vector and VaxGen's AIDSVax has been tested. The vaccine has been shown to be able to induce antibody responses against the second variable (v2) loop of gp 120 of multiple HIV subtypes [66, 67]. This prime-boost vaccine conferred approximately 30% protection against HIV acquisition [68]. The conclusion from the findings of this trial was that the prime-boost vaccine may reduce the risk of HIV acquisition in a community-based population with heterosexual transmission as a main route of HIV transmission. Despite the modest level of efficacy observed by RV 144 trial in Thailand, it provides a platform for future directions in vaccine search by providing evidence that a safe and effective HIV protective vaccine is possible [69].

## 4. Proven New Strategies

### 4.1. Treatment as Prevention (TasP)

Treatment as prevention includes the use of ART in HIV-infected individuals to prevent transmission of HIV to unaffected partners. A study in Uganda demonstrated that there is a significant dose-response relation of increased HIV transmission with increasing viral load [70]. Similar findings were obtained in another meta-analysis; HIV transmission was reduced in patients using ART [71]. It is logical therefore that the lower the viral load, the lower the chances of HIV transmission.

The highest evidence for this is provided by HPTN 052, a large multicontinental trial involving HIV serodiscordant couples [72]. The trial evaluated the impact of early versus late ART initiation in these couples. In this study, it was found out that there was 96% reduction in transmission associated with early ART initiation. The results show that early ART initiation could have the best results in preventing HIV transmission than any other biomedical measures studied to date. The same protective effect could be found in other HIV risk groups such as men who have sex with men (MSM). Adherence to the ARVs has been shown to play a key role in reaching the goal [73]. In view of these findings, several countries in Europe and the regional guidelines recommend the use of early initiation of ART as a strategy to minimize HIV transmission [74]. A cluster-randomized trial (HPTN 071 (PopART)) aiming to test whether widespread provision of ART is feasible and can substantially reduce population-level HIV incidence is currently underway in Zambia and South Africa.

### 4.2. Preexposure Prophylaxis (PrEP)

This is another strategy in which ARVs are used for the prevention of HIV transmission. In this strategy, ARVs are taken before the exposure. The concept of using ARVs as part of preexposure prophylaxis was derived from the same concept used for the prevention of mother to child transmission (PMTCT). Drugs with proven efficacy and safety for use as PrEP include a tenofovir (TDF) and emtricitabine (FTC) coformulation (Truvada).

Evidence for this comes from the iPrEX trial involving a number of men who have sex with men (MSM) in 6 different countries taking daily Truvada [75]. In this trial, men randomized to the Truvada arm were 44% less likely to become infected with HIV than in the placebo arm. The efficacy was further increased with increasing levels of adherence. Those who reported >90% adherence had 68% efficacy while those who reported <50% adherence had only 16% efficacy. Studies in heterosexual partners also using Truvada and tenofovir as PrEP have shown significant reduction in HIV transmission. A study done in Uganda and Kenya involving serodiscordant heterosexual couples showed that couples randomized to tenofovir and Truvada arms were 67% and 75% less likely to become HIV-infected, respectively, compared to those in placebo (Partners PrEP) [76]. The risk reduction was 62.2% in another study done in Botswana using Truvada involving heterosexual couples (TDF-2) [77]. Another trial assessed whether taking a combination of tenofovir and emtricitabine before and after sexual activity (on-demand PrEP) is effective in preventing HIV transmission among men at high risk for HIV-1 infection [78]. Despite an increase in the gastrointestinal and renal side effects, there was a relative risk reduction of 86% in the TDF-FTC group. There is enough evidence to warrant development of guidelines for the use of Truvada for HIV prevention in MSM and serodiscordant couples. However, issues such as development of drug resistance, cost, potential behavioral changes, and risk compensation should not be overlooked.

### 4.3. Male Circumcision

A number of ecological and observational studies have suggested HIV protective effect of male circumcision [79–81]. Since then, several clinical trials have been performed to increase the strength of evidence related to this hypothesis. Three separate trials have demonstrated that male circumcision could reduce male HIV acquisition by up to 60% [82–84]. In addition to HIV, male circumcision also showed protective effects against other STIs such as HPV, HSV-2 [82, 83], and* Mycoplasma genitalium* infections [84]. Male circumcision however has not been shown to reduce HIV to female partners [85]; the same applies for other STIs such as HPV, HSV-2, and* Mycoplasma genitalium* [86, 87]. Several countries in southern and eastern Africa with a high prevalence of HIV and low levels of male circumcision are scaling up male circumcision as part of HIV prevention programs.

### 4.4. Improved Female Condoms

Female-controlled method of protection remains the most promising way to address heterosexual HIV transmission. The female condom remains the only female initiated barrier contraceptive which can protect against HIV, unwanted pregnancy, and other sexually transmitted diseases. Despite worldwide increase in the distribution of female condoms, it remains as the most underutilized reproductive health technology. Evidence suggests that the underuse of female condoms could have been contributed by social stigma and lack of correct information about the product.

The new second-generation female condoms have been developed to improve the acceptability and reduce the cost as well. The first-generation female condoms (FC1) were made from polyurethane. The second-generation female condoms (FC2) are made from synthetic nitrile that diminishes distracting crinkling sounds produced by polyurethane. They have been shown to be effective in the prevention of HIV and other STIs and are available at a lower cost compared with the first-generation female condoms [88]. FC2 have been shown to be a cost-effective method for HIV and other STI prevention even at low adherence [89]. The cost-effectiveness can be further increased by increasing adherence. The protective effect of female condoms for the prevention of STIs and pregnancy has been shown to be similar to that of male condoms [90]. Female condoms could have a more protective effect against syphilis, genital herpes, and human papilloma virus infections because it covers more of the female genitalia than male condoms.

With proper use of the female condom, women can control their own sexual health. The improved female condoms may provide enhanced sensation for men as compared to male condoms; they are hypoallergic and hence minimize the risk for those allergic to rubber latex. It may also be inserted hours before sexual intercourse because they do not depend on an erect penis for insertion. The condom is well lubricated and warms to body temperature. Studies have shown that FC2 performed well in short-term acceptability and crossover studies with FC1 [91, 92]. Some challenges may continue to limit its roll-out, including the higher cost of female condoms compared with male condoms, the need to learn how to properly use the female condom, the distracting noises associated with the original version, and the visibility of the outer ring outside the vagina. Its use also requires some skill that needs to be learned. Also the reported crinkling noises during sexual intercourse in the original version may have put off the potential users. The outer ring of the condom is also visible outside the vagina, another factor that may affect its uptake. However reports show increased update of the female condom with good social marketing and health education [93, 94].

### 4.5. Postexposure Prophylaxis (PEP)

Postexposure prophylaxis is the use of short-term ART to reduce the risk of HIV acquisition following exposure. Existing guidelines recommend initiation of ART within 72 hours after exposure to be used for a total of 28 days [95]. PEP has been used for some time for occupational exposures (since 1990s) and is becoming available for nonoccupational exposures including sex. Evidence for PEP comes from animal studies and retrospective case control analyses of PEP for occupational exposure as well as from the use of ART for preventing mother to child transmission [96–98]. Data regarding the use of PEP following nonoccupation exposure is scarce [99]. Existing evidence suggests that PEP following nonoccupational exposure may be cost-effective, especially in certain population subgroups [99, 100] despite lack of enough evidence on its clinical effectiveness [99].

### 4.6. Mass Drug Administration

There is enough evidence demonstrating that the presence of STIs increases the risk of HIV transmission [101]. Biologically, the assumption is that reducing genital tract inflammation will reduce HIV infectiousness as well as susceptibility in HIV-uninfected individuals. Based on this, treatment of STIs will presumably result in reduction of the risk of HIV transmission. A study done in Mwanza, Tanzania, in mid-1990s demonstrated a 38% reduction in HIV incidence by using a syndromic approach for the treatment of STIs [102]. However, two studies from Uganda [103, 104] and another one from Kenya among female sexual workers [105] did not have similar findings. One of the possible reasons could be the low levels of treatable STIs in Ugandan cohorts [106] and presence of a mature HIV epidemic in Uganda [107]. The successes observed in the Mwanza trial and the benefits from STI programs necessitate the inclusion and integration of STI treatment as part of an HIV/AIDS response [108] despite lack of enough evidence on its benefits.

## 5. Conclusion

HIV prevention remains a constant struggle 30 years since the pandemic began. Focusing on behavioral measures alone has proven to be inefficient. Biological interventions provide new hopes for much more effective HIV prevention. Some of these biomedical interventions have proven efficacy in HIV prevention, while some are still being researched. These new biomedical interventions should therefore be combined with behavioral approaches to maximize the HIV preventive effect. Literature review for this article however was not a systematic review, but rather a minireview that meant to give a synopsis of key papers and studies in the field. A summary of the trials for key papers that summarize these interventions is provided in Table 1.

## Figures and Tables

**Table 1:** Studies on the biomedical technologies and strategies for the prevention of HIV and other STIs.

Biomedical technology/strategy	Study	Study participants	Intervention	Risk reduction
HPV vaccine	—	Heterosexual young men	Bivalent HPV vaccine	91.6% (incident infection)
				100% (persistent infection)

HPV DNA testing	POBASCAM [109]	Heterosexual women	HPV testing	73% relative risk (versus histology)

Improved female condoms	—	Sexually active women	FC2	3% noninferiority margin

Microbicides	CAPRISA 004 [43]		Coital TFV vagina gel	54% (high adherers), 38% (intermediate adherers), 28% (low adherers)

Dapivirine coated vaginal rings	ASPIRE study (MTN 020) [52]	Sexually active women	Antiviral coated vaginal ring	27% risk reduction

HIV vaccine	RV 144 [66]	Heterosexual men and women	ALVAC-HIV and AIDSVAX vaccine regimen (prime-boost vaccine)	31%

TasP	HPTN 052 [110]	Serodiscordant heterosexual couples	Early versus delayed ART treatment	96%

PrEP	iPrEX [75]	MSM	Daily Truvada	44%
	Partner PrEP [111]	Heterosexual men and women	Daily TDF or TDF/FTC	67% for TDF, 75% for TDF/FTC
	TDF-2 [112]	Heterosexual men and women	Daily TDF/FTC	62.2%
	On-demand PrEP [78]	MSM	TDF/FTC	86%
	PROUD [113]	MSM	TDF/FTC	86%

PEP		Health care workers	Zidovudine pill	81%

Male circumcision	ANRS 1265 [82]	Heterosexual men		61% (female-male HIV transmission)

Mass drug administration	Mwanza study [102]	Community	Standardized STI treatment	40% (HIV transmission)
